# Effects of Thymoquinone Alone or in Combination with Losartan on the Cardiotoxicity Caused by Oxidative Stress and Inflammation in Hypercholesterolemia

**DOI:** 10.3390/jcdd9120428

**Published:** 2022-12-01

**Authors:** Ziad H. Al-Oanzi, Fawaz O. Alenazy, Hassan H. Alhassan, Mohamed R. El-Aassar, Abdulaziz I. Alzarea, Sami I. Alzarea, Anass M. Abbas, Muteb H. Alanazi, Maher M. Al-Enazi

**Affiliations:** 1 Department of Clinical Laboratories Sciences, College of Applied Medical Sciences, Jouf University, Sakaka 72341, Saudi Arabia; 2 Department of Chemistry, College of Science, Jouf University, Sakaka 75471, Saudi Arabia; 3 Department of Clinical Pharmacy, College of Pharmacy, Jouf University, Sakaka 72341, Saudi Arabia; 4 Department of Pharmacology, College of Pharmacy, Jouf University, Sakaka 72341, Saudi Arabia; 5 Department of Pharmacy, Prince Sultan Cardiac Center, Riyadh 11159, Saudi Arabia; 6 Department of Medical Laboratory Sciences, College of Applied Medical Sciences, Prince Sattam Bin Abdulaziz University, Al-Kharj 11942, Saudi Arabia

**Keywords:** hypercholesterolemia, thymoquinone, losartan, oxidative stress, inflammation

## Abstract

Dietary cholesterol accelerates oxidative and pro-inflammatory processes, causing hypercholesterolemia and cardiovascular diseases. Thus, the purpose of the current study is to compare the protective effects of thymoquinone (TQ) alone or in combination with losartan (LT) against the heart damage caused by a high-cholesterol diet (HCD). HCD-fed rat groups revealed an elevated activity of indicators of cardiac enzymes in the serum. Serum and cardiac lipids were also found to be significantly higher in HCD-fed rat groups. Cardiac pro-inflammatory and oxidative markers were also increased in HCD-fed rat groups, whereas antioxidant indicators were decreased. However, all of these biochemical, inflammatory, antioxidant, and oxidative change indicators returned to levels similar to those of normal rats after treatment with TQ alone or in combination with LT administered to HCD-fed rat groups. Hypercholesterolemia considerably induced the lipid peroxidation product, thiobarbituric acid reaction substances (TBARs), and oxidative radicals in cardiac cells, which were attenuated by QT and LT treatments, particularly when combined. Finally, QT, LT, and their combination were able to reduce the histological changes changes brought on by cholesterol excess in cardiac tissues. In conclusion, administration of TQ in a combination with LT which has a better protective effect, significantly reduced the hypercholesterolemic-induced oxidative and inflammatory changes that occurred in cardiac tissue.

## 1. Introduction

Increased total cholesterol (TC) levels as well as elevated triglycerides (TG) and low-density lipoprotein (LDL-C) in the circulation are the marks of hyperlipidemia, also known as hypercholesterolemia, appearing as a result of an imbalance in the heart’s metabolic processes, cell signaling, and gene expression. According to past clinical investigations, hypercholesterolemia affects many persons in industrialized nations [1]. It has become common knowledge in the last several decades that increased cholesterol concentrations are associated with an increased risk of cardiovascular disease (CVD) [2]. Extensive clinical investigations indicate that hypercholesterolemia affects a substantial proportion of individuals in industrialized nations [1]. As a direct result of high LDL-C levels, atherosclerosis has become a substantial risk factor for ischemic heart disease, one of the world’s most prevalent causes of death [3]. It has been shown previously that hypercholesterolemia has a direct influence on heart function, which ultimately results in the following impairments: contractile dysfunction [4,5]; exacerbation of ischemia/reperfusion [6]; and decreased response to cardioprotective treatments such as ischemic pre- and post-conditioning [7]. The pathobiology of hypercholesterolemia has been investigated, but the underlying molecular pathways that cause heart problems remain unclear. The condition that is known as hypercholesterolemia, in which there are excessive amounts of cholesterol in the blood, may induce oxidative and nitrative stress in the cardiovascular system. Both of these forms of stress may have a part to play in the onset of heart issues.

The progression of cardiovascular disorders may be mostly attributed to oxidative stress, which is one of the aspects contributing to hypercholesterolemia [8]. Previous research has shown that hypercholesterolemia impairs the action of antioxidants in counteracting the significant rise in reactive oxygen species (ROS) in heart tissue [4,9]. Moreover, it results in endothelial cell activation and increased formation of ROS [10,11], which leads to decreased vascular function, cell death, and remodeling of the heart [12,13]. The use of a high-cholesterol diet (HCD) leads to a rise in oxidative stress, vasoconstriction, and hypertension levels [14]. However, the consequences of an HCD on impaired cardiac function caused by bioenergetics and oxidative stress are not fully understood, and no effective treatment has yet been found. Inflammation is defined as a process associated with oxidative stress, as well as being associated with cardiovascular disease [15,16]. On the other hand, inflammation is defined as a conflict between the body’s oxidative and antioxidant systems, with oxidants acting as the final cause of inflammation [17]. The cell’s internal systems include enzymatic antioxidants, while non-enzymatic defenses [18], including malondialdehyde (MDA), constitute evidence of lipid peroxidation that is used rather frequently [19]. Furthermore, the concentrations of endogenous antioxidants in tissues and enzymatic processes have a significant impact in determining oxidative/nitrosative stress. Nitric oxide (NO) is a powerful antioxidant that is produced by numerous cell types, such as cardiac myocytes. Tumor necrosis factor (TNF-α), Interleukin (IL)-1, and IL-6 are the typical inflammatory cytokines that cause cardiovascular disease [20,21]. TNF-α, IL-1 and IL-6 are among the pro-inflammatory cytokines that are increased in heart failure [22]. Evidence from prior research suggests that TNF-α and IL-6 levels serve as predictors of death in heart failure patients [23,24].

It has been indicated that *Nigella sativa* contains several beneficial chemicals, including thymoquinone (TQ), nigelin, thymohydroquinone, and terpenoids [25,26]. The majority of *N. sativa’s* pharmacological effects are caused by TQ, which is the primary active component. Previous studies have shown that TQ may have an influence on the cardiovascular system [27,28]. For example, the seed of the *N. sativa* plant has been used to successfully treat mild hypertension in human patients [29]. Since the renin-angiotensin system (RAS) is related to the regulation of cardiovascular function, and *N. sativa* inhibits angiotensin-converting enzyme (ACE), it is likely that the antihypertensive effect of *N. sativa* is influenced in part by Ang-II, the principal product of the RAS [30]. A single precursor, angiotensinogen, serves as the basis for the RAS, which then breaks down this precursor to create angiotensin (Ang)I. ACE breaks down Ang I to become Ang II, the RAS’s major bioactive peptide. Angiotensin II (Ang II) is produced by the body using the enzymes renin and ACE, and the AT1 receptor is the primary target for Ang II’s physiological and pathological effects. Hypercholesterolemia-induced vessel damage may be reduced by ACE and AT-1 receptor blockers, according to several studies [31,32,33,34].

Losartan is a first-generation angiotensin-II-receptor antagonist used to treat excessive blood pressure and diabetic nephropathy. Prior research has shown that losartan is better than atenolol for reducing cardiovascular disease and improving blood pressure [35]. Losartan’s therapeutic benefits in cardiovascular events are somewhat effective in decreasing blood uric acid levels, according to a clinical investigation [36]. Several studies have shown increasing interest in the use of several treatments, or poly-therapy. The simultaneous administration of several substances against one or more medical conditions is investigated in this area. Consequently, the objective of this research was to examine the effect of combining QT and LT therapy on the oxidative and inflammatory abnormalities and toxicity seen in HCD-challenged rats.

## 2. Materials and Methods

### 2.1. Animals

King Saud University’s Experimental Animal Care Center provided Wistar albino rats weighing between 150 and 180 g. The animals were provided with controlled conditions such as temperature, humidity, and light (22 + 1 °C, 50–55%, and 12 h light/dark cycles) and spent 7 days getting used to the conditions of the facility before the experiment began. Both Purina rat food and water were readily available to them. According to the Principles and Guidelines for the Care and Use of Laboratory Animals, published by the Institute for Laboratory Animal Research of the National Institutes of Health (NIH Publications No. 80-23; 1996), all procedures, including euthanasia, were carried out with ethical approval (Number: 05-03-42) and guidelines from Jouf University, Saudi Arabia.

### 2.2. Normal Food and HCD Composition

Pellets for a high-cholesterol diet (HCD) were made by mixing powdered 1% cholesterol + 0.5 percent cholic acid with powdered normal cholesterol rat chow (NCRC). Six rats were administered NCRC for six weeks, whereas thirty rats were fed an HCD. For the duration of the experiment, water and food were freely accessible.

### 2.3. Experimental Design

After six weeks of supplementation with NCRC or HCD, the animals were randomly distributed into six groups, with six rats in each group. The first group, the control group of rats, was NCRC-fed with rat chow and was considered the vehicle group. The second group, HCD-treated rats, was considered hypercholesteremic. The third group, HCD-treated rats, was dosed with TQ (10 mg/kg/day). The fourth group, HCD-treated rats, was dosed with LT (10 mg/kg/day). The fifth group of HCD-fed rats received TQ (5 mg/kg/day) + LT (5 mg/kg/day), and the sixth group of HCD-fed rats received atorvastatin (1.0 mg/kg/day). All treatment groups were treated for four weeks. All chemical compounds were supplied from Sigma-Aldrich, St. Louis, MO, USA and given by the College of Pharmacy, King Saud University, SA.

Throughout the duration of the therapy, the HCD diet was maintained. The weekly body weight and general health of the rats were meticulously observed. Whole blood samples were obtained through cardiac puncture under mild ether anesthesia and centrifuged at 4000 rpm for ten minutes. Until analysis, serum samples were kept at −20 degrees Celsius. Finally, the rats were beheaded, and their hearts were removed, weighed, and maintained until analysis at −80 °C. A cross-sectional sample of hearts from each group was kept in 10% formaldehyde for histological analysis.

### 2.4. Characterization of TQ and LT

The morphological topographies of TQ and LT samples were analyzed using a field emission scanning electron microscope (FESEM) (Thermo Scientific Quattro, Waltham, MA, USA). Devices that performed scanning electron microscopy imaging in high-vacuum mode were also utilized. In addition, the structural analysis was evaluated using an ATR-FT-IR spectrometer (a Shimadzu IR–Tracer 100 Fourier Transform Infrared Spectrophotom from Tokyo, Japan), and the Raman spectra of the samples were measured using a Raman spectrometer (HOUND UNCHAINED LABS spectrometer, California, USA). The ^1^H NMR spectra, the ^13^C NMR spectra, and the ^13^C DEPT spectra were all acquired using a JEOL spectrometer with a frequency of 600 MHz (JEOL RESONANCE Inc., Tokyo, Japan).

### 2.5. Serum Analysis

Diagnostic kits were used to determine the concentrations of serum cardiac enzymes, such as lactate dehydrogenase (LDH), creatine kinase-B (CK-B), and creatine kinase-MB (CK-MB) (Human, Germany). Kits manufactured by Randox, London, UK, were used to evaluate lipid profiles (total cholesterol (TC), triglycerides (TG), low-density lipoprotein (LDL) cholesterol (LDL-C), and high-density lipoprotein (HDL) cholesterol (HDL-C). Phospholipid and free fatty acid (FFA) levels in the blood were measured using widely available measuring kits (abcam, Cambridge, UK).

### 2.6. Estimations of Myocardial Lipid Profile

The lipids in the heart were extracted using the [37]) method using a 2:1 (*v*/*v*) mixture of chloroform and methanol. Before centrifugation, tissues were homogenized with 0.74% potassium chloride (1:1 *w*/*v*) and suspended in 2 mL of a chloroform and methanol combination for 2 min. The chloroform layer was dehydrated, and the residual heart lipids were analyzed. The optimal approach of Zilversmit and Davis was used to determine the tissue phospholipid (PL) (1950). Falholt et al.’s technique was used to determine free fatty acids (FFA) (1970). Using commercially available kits (Randox, UK), total cholesterol and triglycerides were calculated. The results were expressed in terms of mg/g of wet tissue.

### 2.7. Estimations of Serum Inflammatory Biomarkers

Using ELISA kits (R&D systems Inc., Minneapolis, USA), the levels of inflammatory biomarkers including IL-6, IL-1 and TNF-α, prostaglandin E-2 (PGE-2), caspase-3 and nitric oxide (NO) were determined.

### 2.8. Estimation of Heart Tissue Oxidative Stress Parameters

Using commercially obtainable ELISA rat kits (Cayman, Michigan, USA), the levels of TBARS and glutathione (GSH) were determined in homogenate from heart tissue, and the post-mitochondrial supernatant of cardiac tissue was analyzed for superoxide dismutase (SOD), catalase (CAT), glutathione peroxidase (GPx) and glutathione-S-transferase (GST)enzyme activity.

### 2.9. Histological Evaluation Method

A piece of cardiac tissue was taken from each group and preserved in 10% formalin. Using a rotary microtome, sections were cut from each sample that had been implanted in a separate paraffin block. Using hematoxylin and eosin, the sections were stained. Finally, the histology was taken by microscope and alterations in histology were analyzed.

### 2.10. Statistical Analysis

The results were expressed as mean and standard deviation. One-way analysis of variance (ANOVA) and the Student–Newman–Keuls multiple comparisons test were used to analyze statistical differences between the groups. If the *p* value is less than 0.05, 0.01, and 0.001, then the corresponding letter and symbol are statistically distinct.

## 3. Results

**Figure 1:**
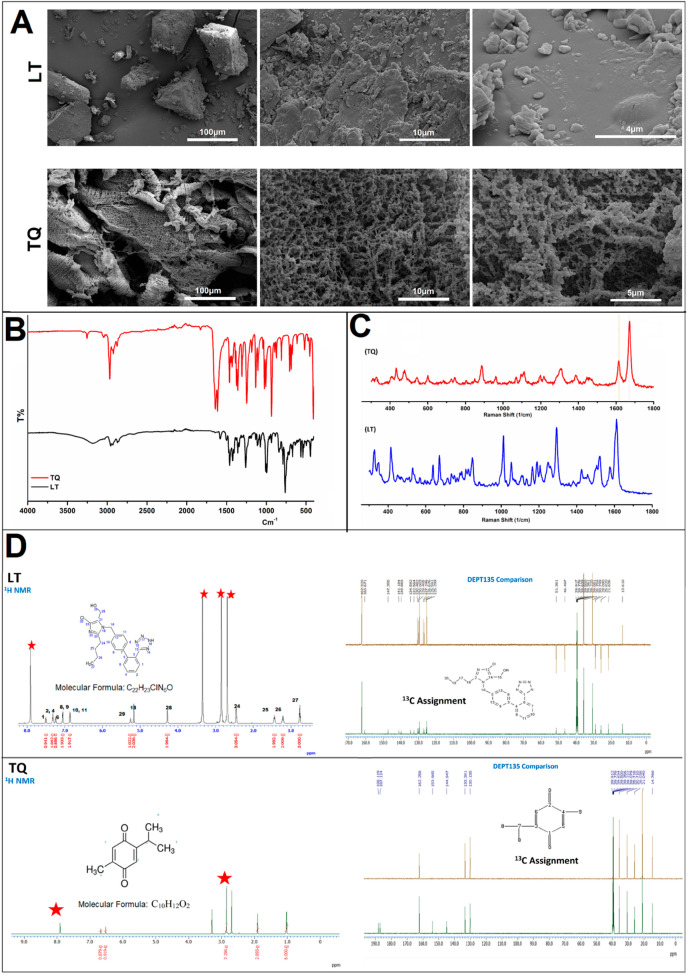
Characterization of thymoquinone (TQ), and losartan (LT) by: (**A**) field emission scanning electron microscope (FESEM). (**B**) ATR-FT-IR spectrometer (**C**) Raman spectrometer. (**D**) Nuclear Magnetic Resonance (NMR) spectroscopy (1H NMR spectra, the 13C NMR spectra, and the 13C DEPT spectra).

TQ and LT are characterized in Figure 1. FTIR, SEM, and NMR spectroscopy were used to establish the structure and purity of TQ and LT. Additional analytical information for the compounds is included in Supplementary Materials section Figures S1 and S2.

**Figure 2:**
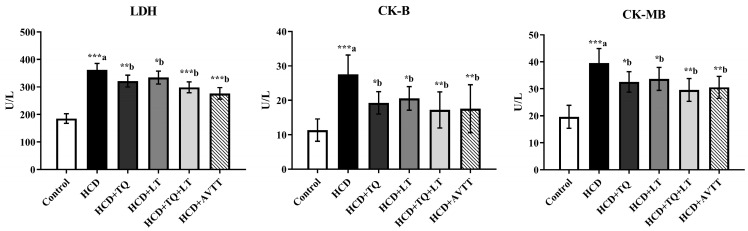
Effect of thymoquinone (TQ), losartan (LT) and their combination on high-cholesterol diet (HCD)-induced changes in serum lactate dehydrogenase (LDH), creatine kinase-B (CK-B) and creatine kinase-MB (CK-MB) levels. Data are expressed as Mean ± SE (*n* = 6) and analyzed using one-way ANOVA followed by Student–Newman–Keuls as post hoc test. ^a^ Control vs. HCD group; ^b^ HCD vs. QT or LT or QT + LT. *p* values considered significant when * *p* < 0.05, ** *p* < 0.01 and *** *p* < 0.001.

Serum levels of cardiac enzymes such as LDH, CK-B and CK-MB were elevated (*p* < 0.001) by more than two-fold in the HCD-fed rat groups as compared to animals fed a normal diet. TQ and LT lowered LDH blood levels in the HCD-fed rat groups (*p* < 0.01 and *p* < 0.05, respectively), when compared to the HCD-fed rat groups. TQ and LT treatments alone decreased LDH blood levels in rats given an HCD, with *p* < 0.01 and *p* < 0.05, respectively, compared to the HCD-fed rat groups. Significant decreases in LDH levels were seen in high-cholesterol rats treated with TQ and LT (*p* < 0.001). Atorvastatin (AVTT), a widely prescribed statin, served as the study’s standard or reference drug. Therapeutically, atorvastatin is used to reduce bad cholesterol and increase good cholesterol. Significantly (*p* < 0.05) decreased serum concentrations of CK-B and CK-MB were seen in the TQ and LT groups compared to the HCD-fed rat groups. In comparison to the HCD-fed rat groups, serum CK-B and CK-MB levels were significantly lower in the TQ and LT-treated groups (*p* < 0.01). CK-B and CK-MB levels were equally suppressed (*p* < 0.01) in rats treated with atorvastatin as they were in hypercholesterolemic animals (Figure 2).

**Figure 3:**
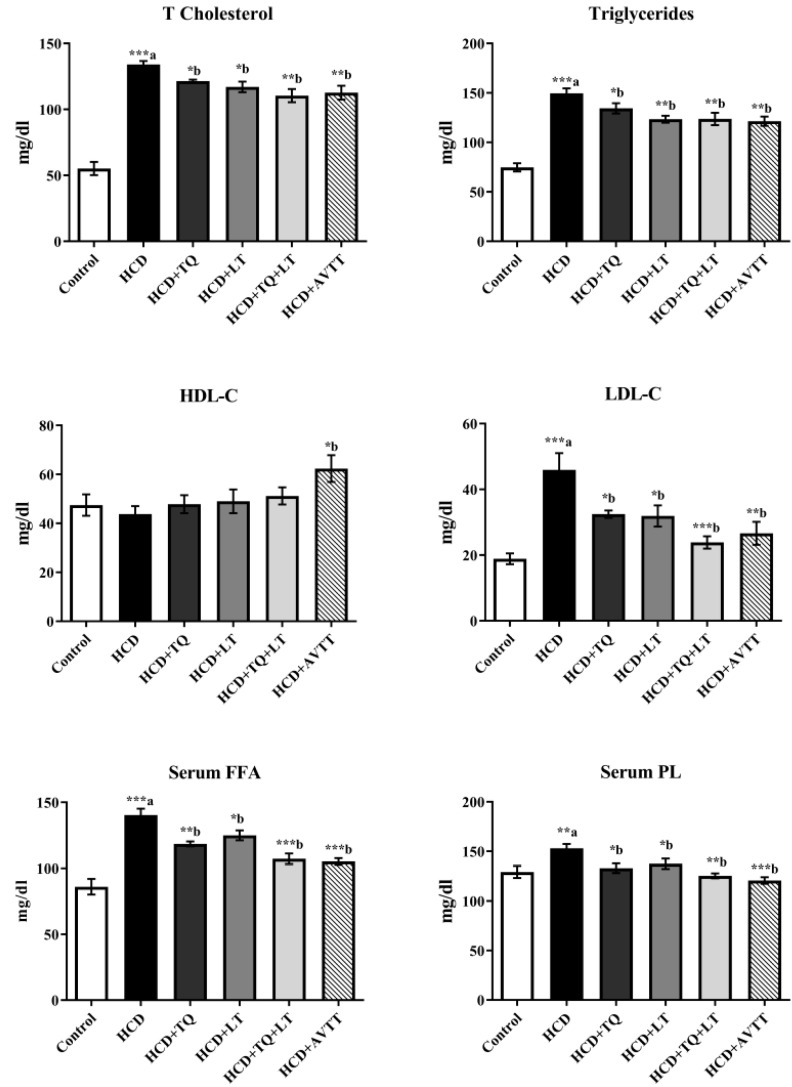
Effects of thymoquinone (TQ), losartan (LT) and their combination on high-cholesterol diet (HCD)-induced changes in serum levels of total cholesterol (TC), triglyceride (TG), high-density lipoprotein cholesterol (HDL-C), low-density lipoprotein cholesterol (LDL-C), free fatty acids (FFA) and phospholipids(PL). Data are expressed as Mean ± SE (n = 6) and analyzed using one-way ANOVA followed by Student–Newman–Keuls as post hoc test. ^a^ Control vs. HCD group; ^b^ HCD vs. QT or LT or QT + LT. *p* values considered significant when * *p* < 0.05, ** *p* < 0.01 and *** *p* < 0.001.

In Figure 3, the blood concentrations of TC, TG, LDL-C and FFA in rats with hypercholesterolemia are shown to be considerably (*p* < 0.001) higher than in normal rats. TQ and LT alone reduced TC serum concentrations in the HCD-fed rat groups at *p* < 0.01 and *p* < 0.05 compared to the untreated HCD-fed rat groups. Combining TQ and LT inhibited TC levels in the blood more effectively (*p* < 0.001) than in the untreated HCD-fed rat groups, and this impact was almost identical to that of atorvastatin, the standard cholesterol-lowering medication. Serum TG levels decreased considerably when rats with hypercholesterolemia were treated with either TQ or LT alone (*p* < 0.05). As a result of the combination of TQ and LT, TG levels in the serum were substantially lower than when they were given separately (*p* < 0.001). The serum TG levels of rats given AVTT were considerably lower than those of rats fed an HCD (*p* < 0.001). The FFA levels of rats given HCD were considerably (*p<* 0.001) higher than those of rats fed a regular diet. The TQ therapy dramatically reduced blood FFA levels compared to the HCD-fed rat groups (*p* < 0.01). FFA levels were also reduced (*p* < 0.05) after treatment with LT. Comparing the TQ and LT groups to the HCD group, blood FFA levels dropped dramatically (*p* < 0.001), but AVTT produced findings that were similar. Phospholipids (PL) in HCD-fed rat groups were considerably (*p* < 0.01) higher than in the normal group. However, individual treatments with TQ and LT significantly (*p* < 0.05) lowered the serum PL levels, whereas the combined (TQ + LT) treated group showed more significant change (*p* < 0.01) when compared to HCD-fed rat groups. Comparing TQ and LT to the HCD-fed rat groups, serum LDL-C values were considerably lower (*p* < 0.01 and *p* < 0.05, respectively). Both TQ and LT showed greater antilipidemic activity than either agent alone, with comparable effects to AVTT. After six weeks of HCD feedings, the serum levels of HDL-C remained almost unchanged throughout the experiments. It was shown that HDL-C levels rose considerably (*p* < 0.05) after treatment with AVTT, in contrast to the HCD-fed rat groups.

**Figure 4:**
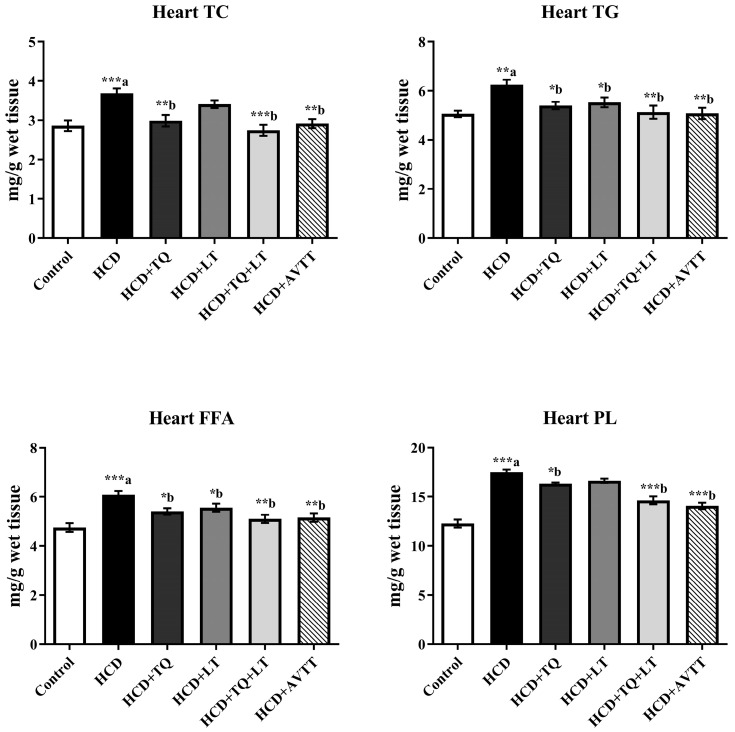
Effect of thymoquinone (TQ), losartan (LT) and their combination on high cholesterol diet (HCD)-induced changes in cardiac levels of total cholesterol (TC), triglyceride (TG), free fatty acids (FFA) and phospholipids(PL). Data are expressed as Mean ± SE (n = 6) and analyzed using one-way ANOVA followed by Student–Newman–Keuls as post hoc test. ^a^ Control vs. HCD group; ^b^ HCD vs. QT or LT or QT + LT. *p* values considered significant when * *p* < 0.05, ** *p* < 0.01 and *** *p* < 0.001.

The cardiac lipid profile is shown in Figure 4. The total cholesterol (TC) levels of rats given an HCD were considerably (*p* < 0.001) elevated, whereas those given TQ therapies were significantly (*p* < 0.01) lower. The combination of TQ and LT considerably (*p* < 0.001) reduced TC levels in cardiac tissues in hypercholesterolemic rats compared to untreated HCD-fed rat groups. Higher amounts of heart tissue TG were seen in HCD-fed rat groups, compared to normal rats (*p* < 0.01). TQ and LT treatment lowered cardiac TG levels in comparison to the HCD-fed rat groups (*p* < 0.05). Nevertheless, cardiac TG levels were higher (*p* < 0.01) in the TQ and LT group than in the AVTT-treated group (*p* < 0.01). Significantly high levels of cardiac FFA were seen in HCD-treated rats compared to normal (*p* < 0.001). TQ and LT therapy significantly reduced myocardial FFA levels in hypercholesterolemic rats (*p* < 0.05) compared to untreated HCD-fed rat groups. The combination treatment of QT and LT was shown to be more effective against hypercholesterolemia, and its results were found to be comparable to those of the AVTT therapy. Rats with hypercholesterolemia had significantly (*p* < 0.001) more phospholipids in their heart tissue than rats with normal cholesterol levels. Cardiomyocyte PL levels were significantly reduced after QT therapy in comparison to untreated HCD-fed rat groups (*p* < 0.05). The combination of TQ and LT reduced myocardial PL levels more significantly (*p* < 0.001) than the HCD-fed rat groups. HCD-fed rat groups given AVTT had substantially lower myocardial PL levels (*p* < 0.001) than untreated HCD-fed rat groups.

**Figure 5:**
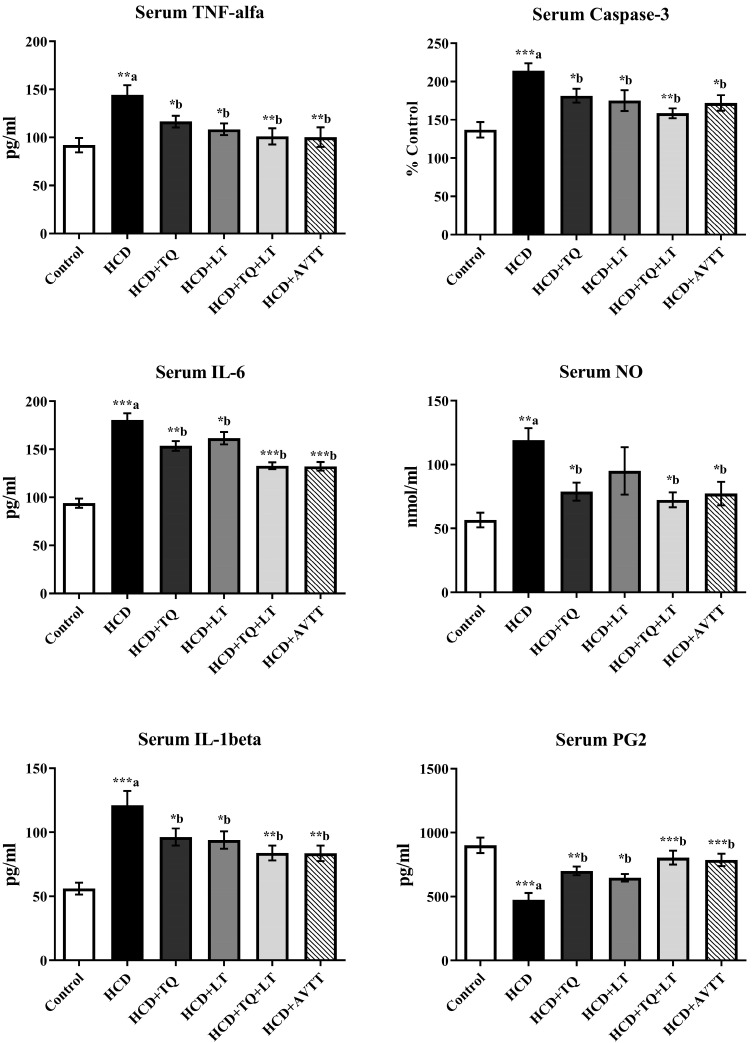
Effect of thymoquinone (TQ), losartan (LT) and their combination on high cholesterol diet (HCD)-induced changes in serum levels of proinflammatory interleukins including tumor necrosis factor-alpha (TNF-alpha), interleukin-6 (IL-6) and interleukin-1beta (IL-1beta) and levels of caspase-3, nitric oxide (NO) and prostaglandin E-2 (PG-2). Data were expressed as Mean ± SE (n = 6) and analyzed using one-way ANOVA followed by Student–Newman–Keuls as post hoc test. ^a^ Control vs. HCD group; ^b^ HCD vs. QT or LT or QT + LT. *p* values considered significant when * *p* < 0.05, ** *p* < 0.01 and *** *p* < 0.001.

Preliminary findings indicate that rats administered HCD showed elevated blood levels of proinflammatory cytokines such as TNF-α, IL-1, and IL-6 (Figure 5). TQ and LT treatment considerably decreased the serum TNF-α levels of hypercholesterolemic rats as compared to untreated HCD-fed rat groups (*p* < 0.05). TQ and LT were more influential (*p* < 0.01) than TQ and LT alone (*p* < 0.05). AVTT-treated rats demonstrated a substantial reduction in TNF-α levels (*p* < 0.01) compared to untreated HCD-fed rat groups. TQ treatment decreased IL-6 levels in the serum considerably (*p* < 0.01) vs. HCD-fed rat groups. Comparing the LT groups to the HCD-fed rat groups, serum IL-6 levels in the LT group were considerably lower (*p* < 0.05). Nevertheless, the combination of TQ and LT significantly decreased IL-6 levels (*p* < 0.001), and AVTT treatment substantially decreased IL-6 levels (*p* < 0.001) compared to untreated HCD-fed rat groups. The serum IL-1β levels of HCD-fed rat groups were considerably (*p* < 0.001) higher than those of normal groups. Treatment of hypercholesterolemic rats with TQ and LT for six consecutive weeks significantly decreased (*p* < 0.05) serum IL-1β levels relative to the HCD-fed rat groups. Compared to the HCD-fed rat groups, the combination of TQ and LT resulted in a more significant (*p* < 0.01) reduction in serum IL-1β levels than TQ and LT alone; AVTT also produced comparable findings.

In Figure 5, high-cholesterol-diet-fed animals’ serum caspase-3 levels were considerably (*p* < 0.001) higher than those of normal groups. It was shown that TQ and LT significantly reduced (*p* < 0.05) the levels of caspase-3 in the serum of HCD-fed rat groups. Comparatively, serum caspase-3 levels were reduced (*p* < 0.01) by the combination of TQ and LT compared to the HCD-fed rat groups. Caspase-3 levels in the serum of AVTT-treated rats were equal to those of untreated HCD-fed rat groups (*p* < 0.05). When compared to normal rats, rats with hypercholesterolemia had considerably (*p* < 0.01) higher amounts of nitric oxide in their serum. Serum NO levels were significantly lower in the TQ treatment groups compared to the HCD-fed rat groups (*p* <0.05). As compared to HCD-fed rat groups, TQ and LT dramatically reduced serum NO levels (*p* < 0.05). Additionally, compared to the HCD-fed rat groups, serum NO levels were lower in those who received AVTT therapy. Serum nitric oxide (NO) levels did not differ substantially between the LT and HCD-fed rat groups. The HCD-fed rat groups showed considerably lower PG2 serum levels than the normal groups (*p* < 0.001). Hypercholesterolemic rats treated with LT for six weeks had a significant increase in serum PG2 levels (*p* < 0.05) compared to the HCD-fed rat groups. TQ treatment raised serum PG2 concentrations in HCD-fed rat groups (*p* < 0.05). On the other hand, rats treated with TQ and LT for six weeks had higher levels of serum PG2 than those in the HCD-fed rat groups. There were significant differences in the HCD groups receiving AVTT therapy, which had raised serum PG2 levels (*p* < 0.001) compared to HCD-fed rat groups.

**Figure 6:**
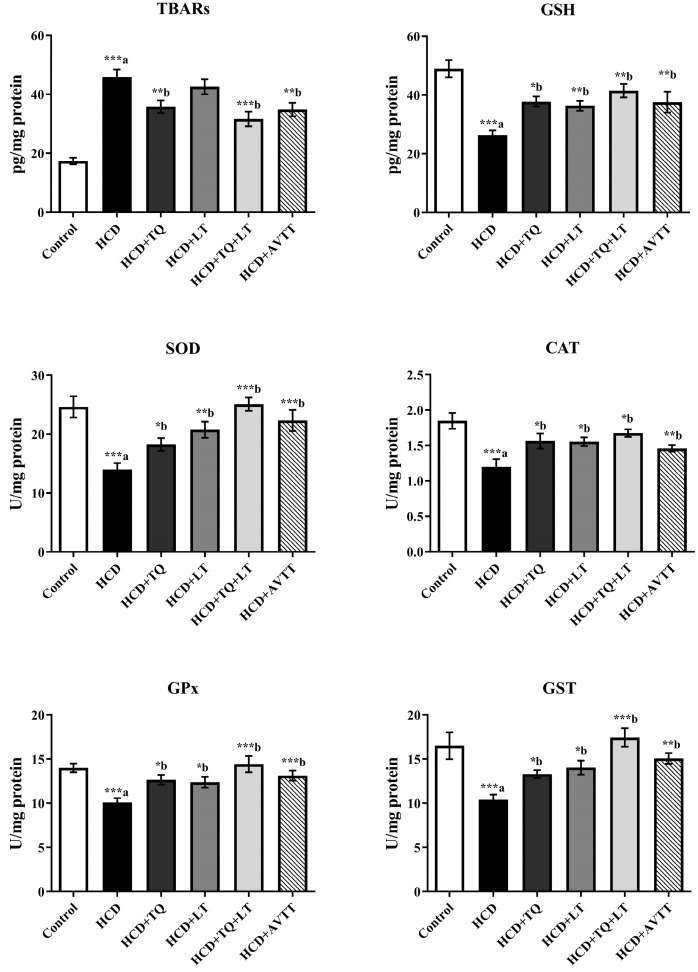
Effect of thymoquinone (TQ), losartan (LT) and their combination on high cholesterol diet (HCD)-induced changes in cardiac tissues levels of oxidative parameters including TBARs, GSH, SOD, CAT, GPx and GST. Data are expressed as Mean ± SE (n = 6) and analyzed using one-way ANOVA followed by Student–Newman–Keuls as post hoc test. ^a^ Control vs. HCD group; ^b^ HCD vs. QT or LT or QT + LT. *p* values considered significant when * *p* < 0.05, ** *p* < 0.01 and *** *p* < 0.001.

The HCD-fed rat groups had considerably higher (*p* < 0.001) levels of heart TBARs, but cardiac cell GSH levels were significantly lower (*p* < 0.001). The TQ therapy significantly reduced the levels of TBARs in HCD-fed rat groups compared to untreated HCD-fed rat groups (*p* < 0.01). When compared to rats treated with HCD, those given TQ and LT had considerably (*p* < 0.001) reduced levels of cardiac TBAR. The AVTT therapy significantly reduced (*p* < 0.01) myocardial TBAR levels as compared to the HCD-fed rat groups. Compared to the HCD-fed rat groups, the TQ and LT groups had substantially higher amounts of GSH in cardiac cells (*p* < 0.05 and *p* < 0.01, respectively). GSH levels were increased (*p* < 0.01) by the combination of TQ and LT. It was shown that AVTT treatment of HCD-fed rat groups significantly increased GSH levels in cardiac cells (*p* < 0.01) compared to untreated HCD-fed rat groups. Compared to the untreated HCD-fed rats, HCD-treated rats had substantially (*p* < 0.001) lower levels of SOD and CAT. SOD activity in cardiac cells was considerably increased in the TQ- and LT-treated HCD group compared to the untreated HCD-fed rat groups at *p* < 0.05 and *p* < 0.01. When compared to untreated HCD-fed rats, the SOD activity in cardiac tissue was significantly (*p* < 0.001) enhanced by the combination of TQ and LT. A substantial increase in myocardial SOD activity was also seen after AVTT therapy (*p* < 0.001). In hypercholesterolemic animals, the QT and LT treatments significantly (*p* < 0.05) increased CAT activity in cardiac cells. QT and LT therapy, as well as AVTT, increased CAT activity in cardiac cells in contrast to untreated HCD-fed rat groups substantially (*p* < 0.05 and *p* < 0.01). In hypercholesterolemic rats, GPx and GST cardiac enzymatic activity was considerably (*p* < 0.001) lower than in normal groups. The enzymatic activities of GPx and GST in cardiac cells were substantially increased (*p* < 0.05) in hypercholesterolemic rats treated with QT and LT compared to untreated HCD-fed rats. However, the combination of QT and LT increased GPx and GST activity considerably in cardiac cells (*p* < 0.001). GPx and GST activities were equally elevated in hypercholesterolemic rats treated with AVTT for six weeks compared to HCD-fed animals not receiving treatment (Figure 6).

**Figure 7:**
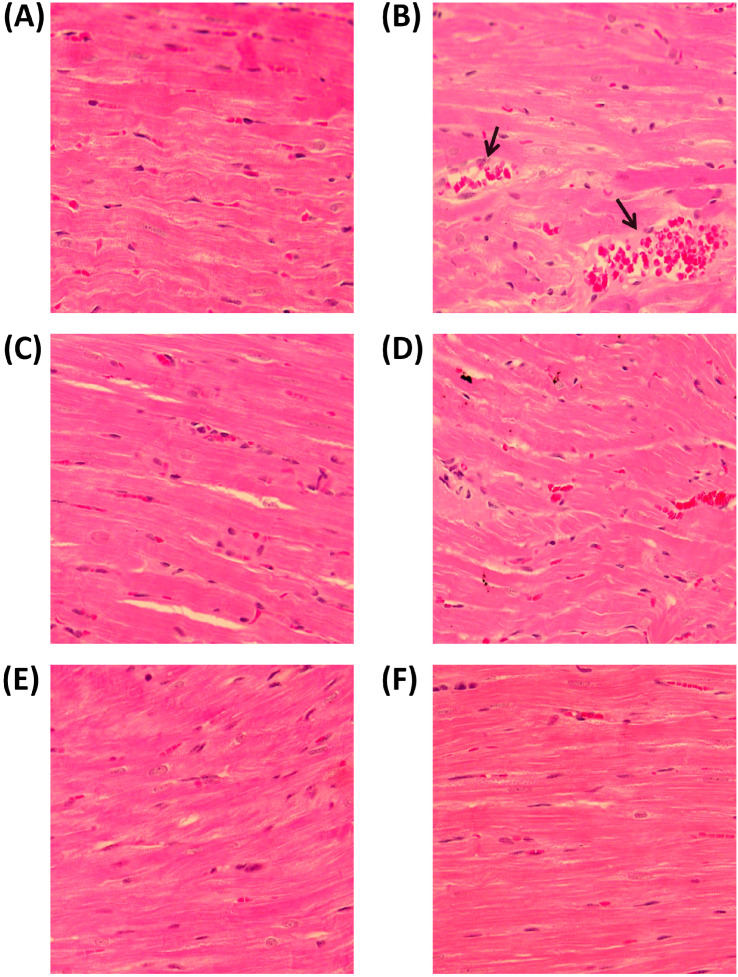
(**A**) Normal control group sections appeared as normal cardiac muscle tissues. (**B**) Hypercholesterinemia revealed focal myocardial stiffening and disarranging, vessel congestion and severe intermuscular inflammatory cell filtration as indicated by the arrow. (**C**) The QT-treated group revealed the appearance of recovered cardiac muscles with mild intermuscular blood congestion. (**D**) The LT-treated group showed cardiac muscles with mild inflammatory cell filtration and mild bleeding. (**E**) The QT and LT combined treatment group did not show any significant changes and had almost normal cardiac tissues. (**F**) AVTT treatment also showed insignificant changes in cardiac tissues, mostly remaining as normal.

The heart tissues of both the control and treatment groups were subjected to histopathological examination. Cross sections of the cardiac muscle of the control group seemed to be in good health (Figure 7A). Cardiovascular congestion, myocardial stiffness and disorganization as well as substantial intermuscular inflammation were all features of hypercholesterolemia (Figure 7B). The appearance of the cardiac muscle in the QT-treated group was restored, with minimal intermuscular blood congestion (Figure 7C). Mild bleeding and moderate infiltration of inflammatory cells were seen in the heart muscles of the LT-treated patients (Figure 7D). Cardiac tissues in the group that received QT and LT in combination showed no significant alterations (Figure 7E). In addition, AVTT therapy has been related to minor changes in cardiac tissues that are mostly benign (Figure 7F).

## 4. Discussion

Hypercholesterolemia is considered to be characterized as abnormally high cholesterol levels in the blood. Diabetes, obesity, genetics, or an unhealthy diet may be underlying causes. LDL cholesterol, also known as low-density lipoprotein cholesterol, is related to an increased probability of atherosclerosis and ischemic coronary artery disease [38]. In addition, preclinical evidence suggests that hypercholesterolemia may seriously affect the heart, leading to dysfunction.

Hypercholesterolemia was seen in HCD-fed rat groups, with TC, TG, and LDL being considerably greater than in control animals. Therefore, high levels of HDL may protect against atherosclerosis by assisting in cholesterol metabolism and removing excess cholesterol from the bloodstream through bile [39]. As a consequence of excessive cholesterol levels, there is a considerable increase in the blood concentrations of CK-B and CK-MB, which cause necrosis and death of cardiac muscle cells. Antioxidant agents have been shown to prevent myocardial necrosis and the redox state in animals with HCD, in addition to their other benefits [9]. Hypercholesterolemia’s detrimental effects on myocardial ischemia–reperfusion deficits may be reversed by AT1R modulation, as research has shown [40]. Additionally, in HCD rabbits, RAS inhibition improved cardiac tissue energy use and blood cholesterol levels [41]. In addition, a tissue study revealed that elevated cholesterol alters the structure of cardiac fibers. According to prior studies, reducing cholesterol improves heart function in hypercholesterolemic animals [42,43]. In terms of cholesterol-lowering supplements, TQ has become a popular choice. More than a dozen studies have shown that it lowers LDL and raises HDL, indicating that it is effective in the prevention of hypercholesterolemia and the consequences of atherosclerosis [44]. New Zealand white rabbits suffering from hypercholesterolemic atherosclerosis were treated with TQ, which reduced the levels of lipids in their blood [45]. According to available clinical and experimental evidence, the favorable effects of TQ may go beyond cholesterol reduction to include what are referred to as multidirectional effects. The results show that in HCD-fed rat groups, TQ lowered the blood levels of lipid profiles of TC, TG, and LDL. In hypercholesterolemic rats, we compared the antihypertensive and antihypercholesterolemic effects of TQ and LT, respectively. The findings of the current study reveal that treating hypercholesterolemic rats with LT and TQ for many weeks normalized their blood lipid profile. The combination of TQ and LT has also been shown to have a better likelihood of preventing mice from developing high-cholesterol-related heart disease.

According to the findings of prior research, oxidative stress is important in many different types of chronic diseases, as well as cardiovascular disease, diabetes, malignancy, kidney, neurological disorders and inflammatory-related diseases. It is affected by a defect in the production and buildup of reactive oxygen species and reactive nitrogen species (ROS/RNS), which leads to high concentrations of these species in tissues and cells due to the body’s inability to reduce them [46]. The reactive oxygen species (ROS) that are formed in chronic disorders are mostly generated by a family of non-phagocytic nicotinamide adenine dinucleotide phosphate (NADPH) oxidases, which includes the prototypical Nox2-based NADPH oxidases, Nox1, Nox4, and Nox5. Antioxidant enzymatic systems include SOD, CAT, GPx, and GST; other probable sources include enzymes involved in the transfer of electrons inside mitochondria, as well as xanthine oxidase, cyclooxygenase, lipoxygenase, and uncoupled nitric oxide synthase (NOS) [47]. Decreased endogenous antioxidant capability might be another potential cause for increased oxidative stress. In reality, hypercholesterolemia has been linked to lower levels of antioxidant enzyme production and cardiovascular activity [48,49,50]. However, the specific molecular processes through which elevated cholesterol inhibits myocardial antioxidant enzymes are unclear. Aside from enzymatic activities, tissue levels of endogenous antioxidants are important in defining oxidative and/or reactive stress. NO synthases in various cells, including cardiomyocytes, produce this vital antioxidant molecule. In addition, the blood levels of caspase-3 and NO in HCD-fed rats were considerably elevated. These chemicals are used to detect cellular injury and apoptosis. Hypercholesterolemia has been proven in studies to cause apoptosis [51] and the production of NO [52].

In the current study, many markers of oxidative stress were examined in various kinds of cardiac tissue. Lipid peroxidation and reduced glutathione peroxidase (GSH) levels result from the oxidative stress caused by hypercholesterolemia. In addition, animals receiving HCD had reduced antioxidant enzyme activity, which protects cells from oxidative injury produced by reactive oxygen species (ROS). During the first stages of oxidative stress, the expression and activity of antioxidant enzymes increase. This is due to the fact that antioxidant enzymes safeguard cellular activities [53]. According to replications of this research, the activation of antioxidant genes seems to be driven by free radicals. However, chronic oxidative stress reduces the activity of these antioxidant enzymes. Possible explanations for this include “defense fatigue,” in which cells are unable to manufacture these enzymes because they are constantly and vigorously attacked by free radicals. Moreover, antioxidant enzymes are susceptible to oxidation by free radicals [53]. Furthermore, it is well recognized that monocytes are driven to produce systemic inflammatory cytokines in the presence of hypercholesterolemia [54]. Due to the significance of these inflammatory mediators in the pathogenesis of several metabolic syndromes, they may be used as part of a diagnostic panel for cardiovascular and metabolic illnesses. Ingestion of a HCD dramatically elevated blood levels of IL-1, IL-6, TNF-α, and PGE2 in the current investigation, indicating a high level of systemic inflammation. Our success in finding support for the findings of Chan and colleagues indicates that experimental hypercholesterolemia is linked to decreased antioxidant capacity and systemic inflammation [55]. Furthermore, the levels of caspase-3 and NO in the blood of HCD-fed rat groups increased significantly. These substances serve as indicators of cellular damage and the apoptotic process. Hypercholesterolemic rats may exhibit elevated systemic inflammation and apoptosis as a consequence of oxidative and nitrosative cell damage, which promotes leukocyte trafficking and increases vascular permeability. This is possible given that both nitrosative and oxidative stress induce cell damage [56]. In addition, RAS has been linked to systemic inflammation, which reflects an elevated blood cholesterol level. It has been shown that Ang II promotes an increase in monocyte migration and pro-inflammatory cytokines in individuals with hypercholesterolemia [57]. Additionally, it is known that Ang II may both increase and control ROS and NO production [58]. In contrast, limiting the last phase of the Ang II cascade by decreasing AT1R may lead to a significant depletion in mediators of systemic inflammation and caspase-3. This was seen in mice administered LT in the present investigation. Similarly, IL-1, IL-6 and TNF-α significantly decreased the high levels of pro-inflammatory cytokines. Our findings agree with past in vivo and vitro studies [59,60].

According to previous research, TQ may exercise its antioxidant properties by reducing the activity of reactive oxygen species (ROS) and improving the movement of other molecules capable of scavenging radicals. Experimental rabbits showed that TQ had a curative impact on atherosclerosis caused by hypercholesterolemia and reduced oxidative stress levels [45]. Over an eight-week period, TQ reduced cholesterol-induced elevations in blood and aortic tissue MDA concentrations [61]. Ahmad and Beg confirmed TQ’s anti-atherosclerotic properties in a second in vivo study [62]. Antioxidant activity is further supported by the fact that TQ increased the levels of SOD (a powerful radical scavenger) in the bloodstream, which is consistent with Nemmar and Al-Salam (2011) and others. Second, in vivo studies by Liu and colleagues establish that TQ reduces the severity of diabetes-induced cardiovascular disease (CVD) in male Wistar albino rats. MDA levels were reduced while SOD enzyme levels were increased, indicating heightened antioxidant and radical scavenging capability. This was accomplished by elevating SOD enzyme levels [59]. TQ’s antioxidant activities were further studied in a second in vivo experiment on male rats [63]. In rats that were given a diet high in cholesterol, administration of TQ at dosages of 20, 50, and 100 mg/kg per day enhanced both antioxidant efficacy against hydroxyl radicals and the mRNA expression of the antioxidants CAT, SOD, and GPx. According to our findings, TQ could be a useful radical scavenger. Oxidative stress markers such as TBARS were reduced by TQ administration, whereas GSH levels were elevated, and the enzyme activity of SOD, CAT, and GPx was enhanced in the current research. Oxidative damage to the heart muscle may be prevented by increasing glutathione levels, stimulating antioxidant enzymes, and reducing lipid peroxidation, according to the findings of this research.

## 5. Conclusions

In conclusion, TQ was able to prevent the oxidative damage, inflammation, and necrosis found in hypercholesterolemic rats, either on its own or in combination with LT. However, the findings suggested that the combined therapies had a considerable synergistic protective effect. These benefits may also be the result of the activation of cellular antioxidant enzymes and/or the regulation of inflammatory cytokines, as well as the restoration of tissue-regulating histological characteristics and the abatement of cardiac oxidative stress.

## Data Availability

All data collected in the manuscript are available upon request.

## Supplementary Materials

The following supporting information can be downloaded at: https://www.mdpi.com/article/10.3390/jcdd9120428/s1. Figure S1: SEM micrographs of the surface of (A) Losartan (LT), and (B) Thymoquinone (TQ), and (A*, B*), LT and TQ elemental distribution by EDX; Figure S2: The chemical structures of (A) Losartan (LT), and (B) Thymoquinone (TQ) used in this work.
