# Supplementary material for: Effects of Thymoquinone Alone or in Combination with Losartan on the Cardiotoxicity Caused by Oxidative Stress and Inflammation in Hypercholesterolemia
Source: J Cardiovasc Dev Dis. 2022 Dec 1;9(12):428. doi: 10.3390/jcdd9120428 (PMC9782872; doi:10.3390/jcdd9120428)
Supplement: Supplementary file 1 [file jcdd-09-00428-s001.zip › jcdd-2045569-supplementary.pdf]

Supplementary material

1. SEM-EDX Analysis

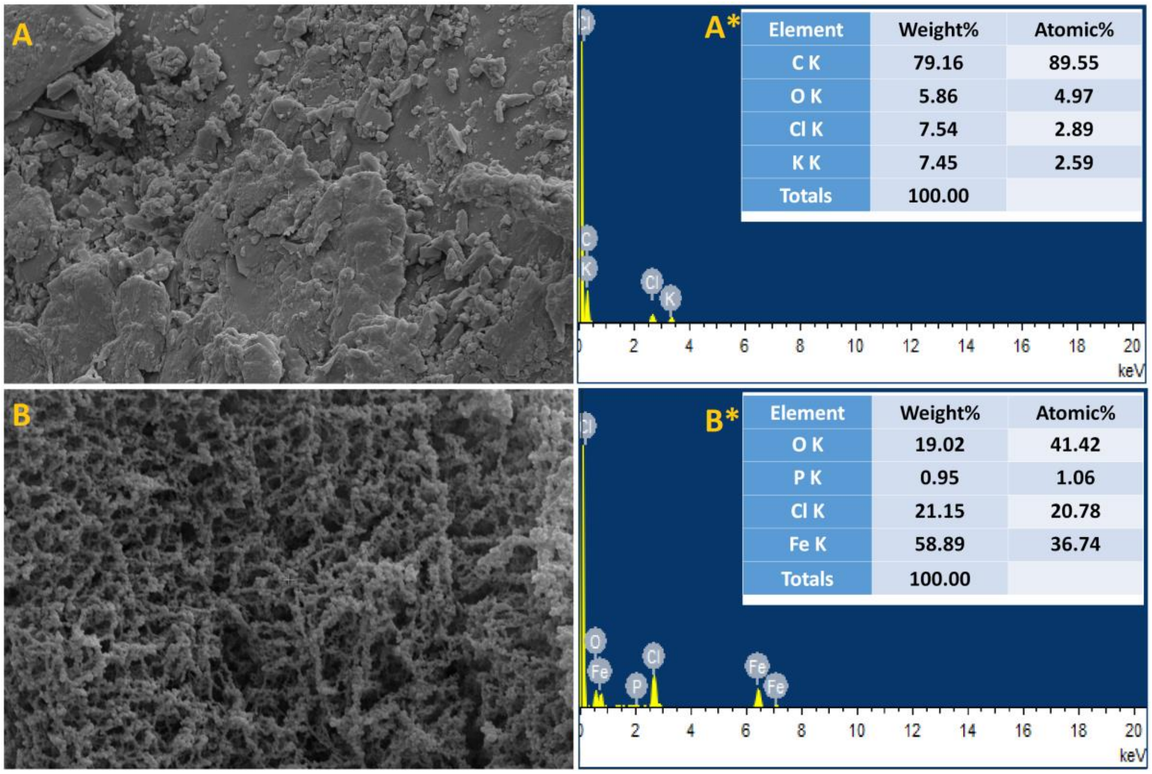

Figure S1: SEM micrographs of the surface of (A) Losartan (LT), and (B Thymoquinone (TQ), and (A*, B*), LT and TQ elemental distribution by EDX

2. NMR Signal Assignment (TQ & LT)

(A) Losartan (LT)

Molecular Formula: $C_{22}H_{23}ClN_6O$

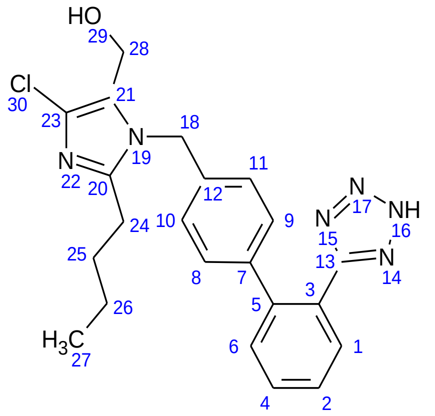

^{1}H Assignment

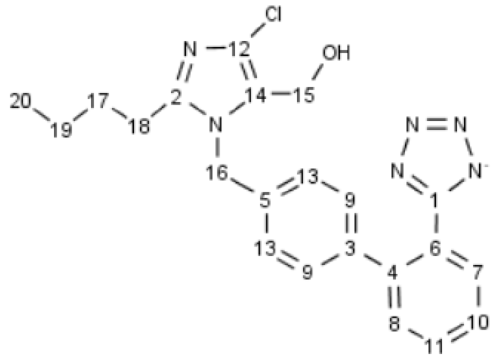

^{13}C Assignment

(B) Thymoquinone (TQ)

Molecular Formula: $C_{10}H_{12}O_2$

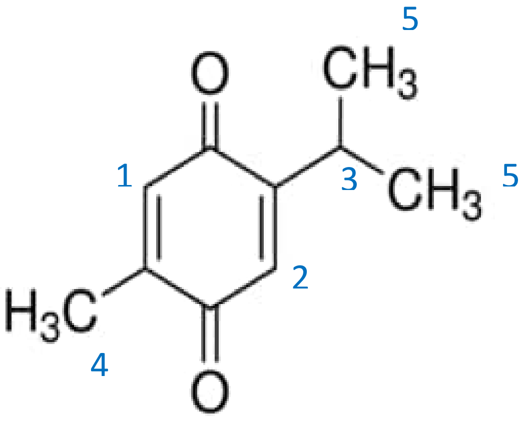

^{1}H Assignment

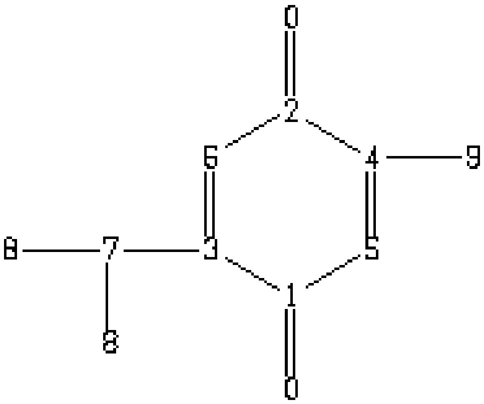

^{13}C Assignment

Figure S2: The chemical structures of (A) Losartan (LT), and (B Thymoquinone (TQ) used in this work.

Losartan (LT) Assignement

	13C-Signal	Assignement	DEPT Assignment
1	162.3203	Unwanted/Impurity	Unwanted/Impurity
2	160.671	1	Quaternary Carbon
3	147.3549	2	Quaternary Carbon
4	141.184	3	Quaternary Carbon
5	139.8627	4	Quaternary Carbon
6	134.5918	5	Quaternary Carbon
7	132.5643	6	Quaternary Carbon
8	130.4818	7	-CH
9	130.0031	8	-CH
10	129.4046	9(two Carbons)	-CH
11	127.2455	10	-CH
12	126.6758	11	-CH
13	125.5915	12	Quaternary Carbon
14	125.2995	13(two Carbons)	-CH
	125.285	14	Quaternary Carbon
15	51.36074	15	$-CH_2$
16	46.49676	16	$-CH_2$
17	39.9165	Solvent Peak	NA(Not Applicable)
18	39.77767	Solvent Peak	NA
19	39.63883	Solvent Peak	NA
20	39.5	Solvent Peak	NA
21	39.36117	Solvent Peak	NA
22	39.21994	Solvent Peak	NA
23	39.0811	Solvent Peak	NA
24	35.75866	Unwanted/Impurity	Unwanted/Impurity
25	30.74867	Unwanted/Impurity	Unwanted/Impurity
26	29.08984	17	$-CH_2$
27	25.82006	18	$-CH_2$
28	21.63589	19	$-CH_2$
29	13.60984	20	$-CH_3$

^{13}C Signal Assignment

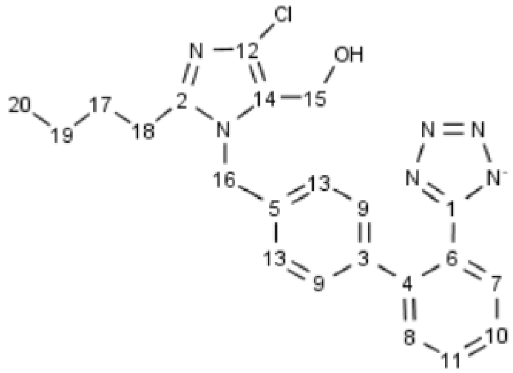

^{1}H Signal Assignment

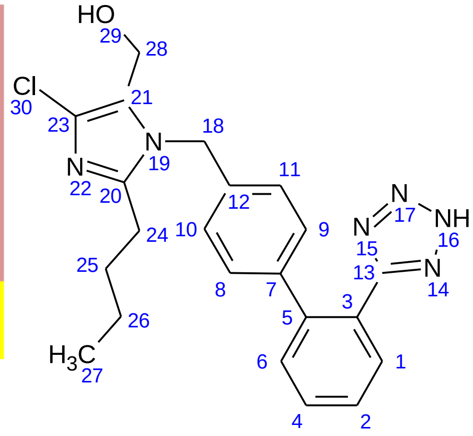

Thymoquinone (TQ) Assignment

Sl. No.	^{13}C Chemical Shift	^{13}C Assignment	DEPT Assignment
1	188.119[ppm]	1	Quaternary Carbon
2	187.114[ppm]	2	Quaternary Carbon
3	162.258[ppm]		Unwanted/Impurity
4	153.985[ppm]	3	Quaternary Carbon
5	144.947[ppm]	4	Quaternary Carbon
6	133.381[ppm]	5	-CH
7	130.135[ppm]	6	-CH
8	39.912[ppm]		Solvent Peak
9	39.778[ppm]		Solvent Peak
10	39.634[ppm]		Solvent Peak
11	39.500[ppm]		Solvent Peak
12	39.356[ppm]		Solvent Peak
13	39.222[ppm]		Solvent Peak
14	39.079[ppm]		Solvent Peak
15	35.718[ppm]		Unwanted/Impurity
16	30.710[ppm]		Unwanted/Impurity
17	26.038[ppm]	7	-CH
18	21.040[ppm]	8(02- Carbons)	CH_3
19	14.768[ppm]	9	CH_3

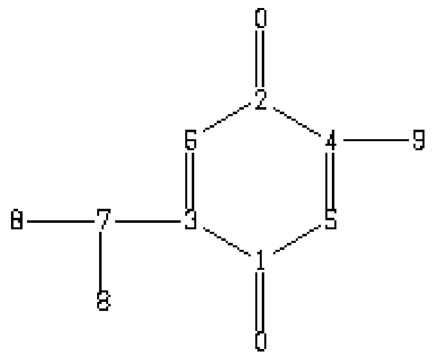

*Red highlighted rows are impurities or unwanted peaks
